# Low bend loss femtosecond laser written waveguides exploiting integrated microcrack

**DOI:** 10.1038/s41598-021-03116-y

**Published:** 2021-12-09

**Authors:** Timothy Lee, Qi Sun, Martynas Beresna, Gilberto Brambilla

**Affiliations:** Optoelectronics Research Centre, University of Southampton, Southampton, SO17 1BJ UK

**Keywords:** Laser material processing, Integrated optics

## Abstract

We introduce the fabrication and use of microcracks embedded in glass as an optical element for manipulating light propagation, in particular for enhancing waveguide performance in silica integrated optics. By using a femtosecond laser to induce a strong asymmetric stress pattern in silica, uniform cracks with set dimensions can be created within the substrate and propagated along a fixed path. The smoothness of the resulting cleave interface and large index contrast can be exploited to enhance waveguide modal confinement. As a demonstration, we tackle the longstanding high bend-loss issue in femtosecond laser written silica waveguides by using this technique to cleave the outer edge of laser written waveguide bends, to suppress radiative bend loss. The microcrack cross section is estimated to be 15 μm in height and 30 nm in width, for the 10 $\times$ 10 μm waveguides. At 1550 nm wavelength, losses down to 1 dB/cm at 10 mm bend radius were achieved, without introducing additional scattering. Both the cleave stress pattern and waveguide are fabricated with the same multiscan writing procedure, without requiring additional steps, and re-characterisation of the waveguides after 1 year confirm excellent long term performance stability.

## Introduction

Strong research interest in ultrafast laser material processing has been motivated by the ability to modify refractive index^1^, machine or ablate^2,3^, and nanostructure^4–7^ with excellent resolution all using a single system, with a host of applications in the fabrication of low-cost optical chips such as quantum circuits^8–11^ and integrated biophotonics^12,13^. In particular, direct laser writing of such optical chips by focusing femtosecond pulses into transparent media, wherein nonlinear effects including multiphoton absorption and avalanche lead to a localised and permanent index change $\Delta n$ at the focal volume^14^, allows the fabrication of embedded waveguide devices^1,15–17^ with submicron feature size and ~10 nm resolution. However, one fundamental limitation is the low induced $\Delta n$, typically $10^{-3}$ in fused silica, which restricts optical functionality of the resulting devices. While it is possible create a high index-contrast by inducing voids via microexplosions^18^ and also by dicing the glass to create an interface with air^19^, the former is highly scattering and the latter cannot be locally integrated into devices. To overcome this shortcoming, we introduce and demonstrate an additional processing technique to locally cleave the glass: by focusing femtosecond laser pulses into a glass substrate to induce an appropriate stress pattern, we can create and accurately guide the localised formation of microcracks, i.e. cracks with a typical cross section height of the order of 10 μm or more and a submicron width. To date, cracks are often considered as damage during laser inscription and parameters are chosen specifically to avoid their formation, whether for optical^20^ or mechanical structures^21^. However, contrary to this common notion, we demonstrate that it is possible to create mechanically stable cracks with optically smooth non-scattering interfaces (indeed, even for macroscopically stress-diced glass the surface roughness is on the order of only 10 nm^22^), cleaved along pre-defined 3D paths embedded in the glass and thus confined with precise dimensions and position.

The interface smoothness, large index contrast ($\Delta n \approx 0.45$ in silica) and compactness of the microcrack can all be exploited for integration with photonic structures to help overcome constraints related to low refractive index change during laser writing. To demonstrate an application for improving photonic circuits, we selectively created cracks on the outer bend edge of femtosecond laser written curved waveguides to reduce bend loss by utilising the strong index contrast to enhance modal confinement. This endeavour addresses a critical issue hampering development of compact femtosecond laser written photonic circuits—high bend loss $\alpha _b$ due to low $\Delta n$, which limits the minimum usable bend radius $r_b$ to a few cm^23^. Efforts to reduce $\alpha _b$ so far include annealing to improve the index profile^16^ and writing neighbouring stress structures^24^, but both methods require additional time consuming steps, and $\alpha _b$ still remains notably higher than that of regular SMF-28 fiber. Our approach is simpler yet highly effective, as the waveguide inscription also simultaneously induces the stress pattern for crack formation, and benefits compact multi-waveguide designs as the crack does not occupy additional volume.

**Figure 1:**
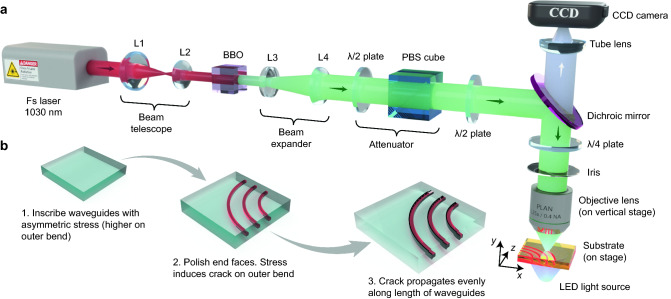
Experimental set up and fabrication procedure. (**a**) Femtosecond writing system for inscription of waveguides. The focusing objective is mounted on a vertical stage to adjust height, and the silica substrate is placed on a two-axis translation stage. (**b**) Procedure for fabricating microcrack along outer edge of curved waveguides.

## Results

### Induced asymmetric stress profile

To produce our waveguides and cracks, a standard femtosecond laser writing system illustrated in Fig. 1a was used and the procedure is summarised in Fig. 1b. The waveguides were formed by multiscan, i.e. rastering closely spaced scanlines (see “Methods” section) using a focused Gaussian beam. Importantly, scanline order is known to influence the stress pattern^25^, and successive adjacent scanlines will accumulate stress if their lateral separation is narrow enough and pulse energy or pulse density are sufficiently high, producing an asymmetric stress profile with a non-central peak skewed towards the final scanline. This peak stress is chosen high enough for crack propagation but not spontaneous formation (a less predictable process). To induce the crack, at least one end of the waveguide is lapped, during which surface flaws acting as stress concentrators cause crack nucleation, followed by propagation via a ‘weakest-link’ model^26^ favouring the highest stress path induced by the multiscan. This exploits the fact that while Si–O bonds are intrinsically strong and the theoretical elastic limit of fused silica is high (at least 15 GPa), in practice fracture occurs at only of tens of MPa^27^. Consequently, its fracture toughness $K_{Ic}$ of 0.5 to 1 MPa m$^{1/2}$ is low which indicates weak resistance against crack propagation^28^.

**Figure 2:**
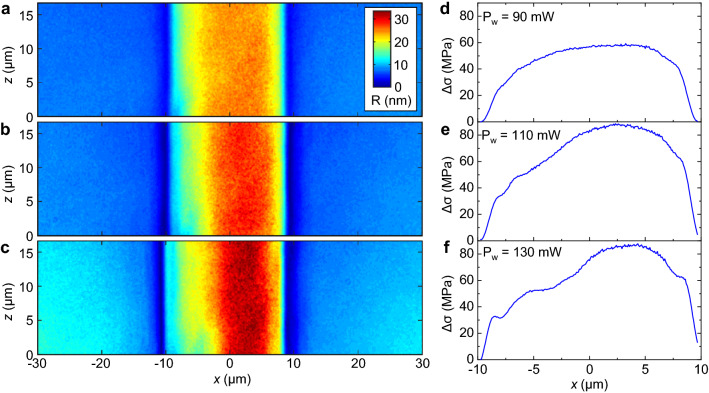
Asymmetric retardance and principal stress-difference profiles of structures fabricated by multiscan. Top down retardance profiles *R* of multiscanned waveguide-like structures written by Gauss-Bessel beam at writing powers $P_w$ of (**a**) 90 mW, (**b**) 110 mW and (**c**) 130 mW ($\pm 0.05$ mW). Waveguide width 20 μm. Scanlines are parallel to the *z* axis, with first scanline on the left-most side. (**d**–**f**) The corresponding principal stress difference profiles $\Delta \sigma$, averaged in the *z* direction.

To verify that the stress profile induced by a multiscan is indeed asymmetric, we performed retardance and stress profile measurements. For the purpose of these tests, we wrote straight waveguide-like structures ($\approx$ 20 $\times$ 100 μm cross section) at different powers $P_w$ using a Gauss-Bessel beam. Such Gauss-Bessel beams are often used when processing larger depths or volumes of material quicker^29^. The use of a larger structure for these tests allows for easier imaging and characterisation with the microscope on which the measurements are taken. After fabrication, their retardance profiles *R* in Fig. 2a–c were measured to determine their principal stress difference profiles $\Delta \sigma$ in Fig. 2d–f. Each waveguide was 20 μm in width and the multiscan began from the left side; the retardance and stress profiles skew to the right due to stress accumulation. Asymmetry is minimal at a low writing power of $P_w=90$ mW, but grows significantly for higher powers: at 130 mW the stress difference at $x=+5$ μm is 85 MPa, 70% greater than at $x=-5$ μm.

### Simulations

**Figure 3:**
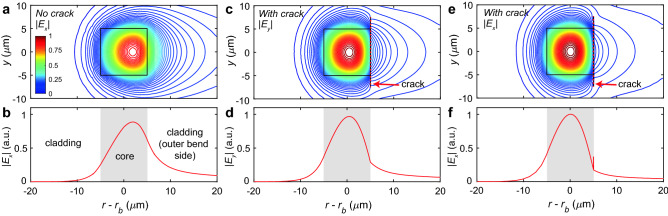
Simulated effect of crack on modal confinement in curved waveguides. (**a**, **b**) Fundamental mode electric field profile and cutline across $z=0$ for the case $r_b=7$ mm without crack for *x* polarization, (**c**, **d**) with crack for *y* polarization and (**e**, **f**) with crack for *x* polarization. Waveguide is 10 $\times$ 10 μm; crack is 30 nm wide and 15 μm high located on outer bend edge of waveguide indicated by dotted red line; $\lambda =1.55$ μm. Shaded grey region in cutline plots indicates extent of core.

To illustrate the mechanism by which the microcrack reduces bend loss, we simulated the $\lambda =1.55$ μm fundamental mode field profiles of a curved waveguide as shown in Fig. 3, for a $10\times 10$ μm core silica waveguide of bend radius $r_b=7$ mm, with and without a crack on the outside bend edge of the core. The core-cladding index difference was taken as $4.1\times 10^{-3}$, based on previous experimental measurements, and crack width was estimated as $w_c=30$ nm from comparisons of the simulated results for different widths. In Fig. 3a, b, the waveguide without the crack is poorly guiding with a high radiative loss, as can be seen by the significant portion of the field to the right of the waveguide. The cutline also shows the mode peak skewed toward the outer bend direction, rather than centered on the waveguide. Since the core-cladding index here is weak, the *x* and *y* polarization mode profiles are almost identical, with a calculated bend loss of 6.5 dB/cm.

When a crack is introduced, the mode profiles in Fig. 3c–f confirm that the microcrack helps to suppress the radiated field strength on the outer bend region, and the mode peak more centered on the core. The calculated bend losses are reduced to 2.1 dB/cm and 3.9 dB/cm for the *x* and *y* polarizations, respectively. The polarization dependent loss (PDL) arises because while both polarizations experience much tighter modal confinement (and hence lower radiative loss) than the uncracked case, the *x* polarization benefits greater due to its field being perpendicular to the high index contrast interface; a feature generally seen in strongly guiding waveguide systems^30^.

### Microcrack fabrication and characterisation

**Figure 4:**
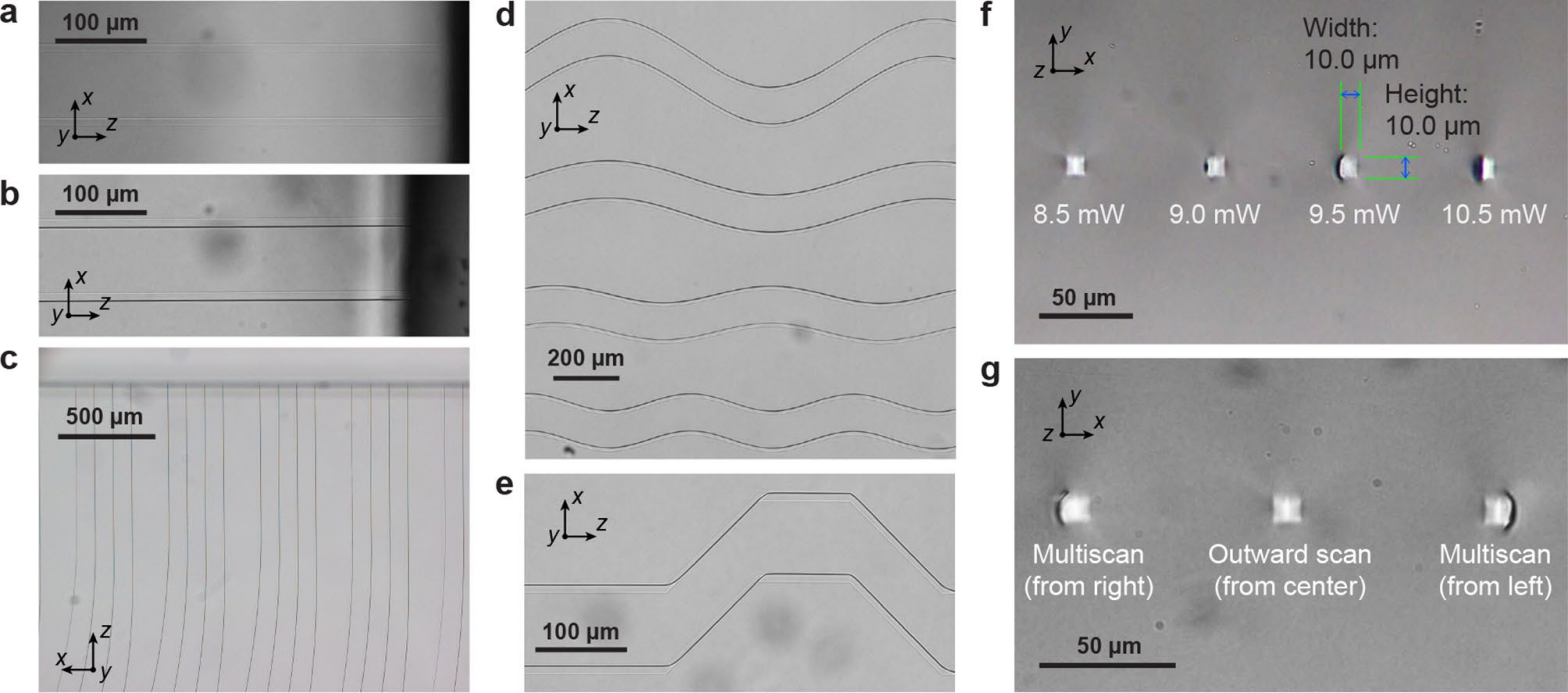
Bright field microscopy of fabricated waveguides and integrated cracks. (**a**) Top-down images of waveguides before polishing and (**b**) after polishing, with cleaved microcrack visible on lower edge. (**c**) Waveguide bend arrays. (**d**) Examples of cracks fabricated along sinusoidal and (**e**) stepped 45$^\circ$ angle paths. (**f**) Cross sections of waveguides inscribed at writing powers $P_w$ from 8.5 to 10.5 mW ($\pm 0.05$ mW), and (**g**) using different multiscan line orders, for $P_w$ = 9.5 mW. All waveguides written with width 10 μm, pulse density $D=3\times 10^5$ pulses/mm, scanline spacing $s=200$ nm, $\lambda _w = 515$ nm, 0.4 NA objective lens, and circular polarization.

Next, we performed experimental confirmation by fabricating $10\times 10$ μm cross section waveguides of bend radii in the range $r_b=3$ to 15 mm over a 90$^{\circ }$ angle in fused silica. These were inscribed using a $\lambda =515$ nm focused Gaussian writing beam with 200 fs pulses at 200 kHz repetition rate using the system in Fig. 1 (details in “Methods” section). A multiscan (beginning from the inner bend) inscribed both the core and the asymmetric stress profile defining the crack path simultaneously, i.e. the stress induced by writing the waveguide is already asymmetric and sufficiently high for the crack to propagate during subsequent lapping. To inhibit nanograting damage which exacerbates scattering and PDL^31^, a circularly polarized writing beam was used. The pulse density along the scanlines was unusually high at $D=3\times 10^5$ pulses/mm to induce stronger stress and index change, as well as improve crack uniformity.

Microscope images in Fig. 4a, b confirm successful cleaving of the outer bend edge of the waveguides after lapping/polishing, clearly visible as a dark edge due to the microcrack $\Delta n$ of 0.45 being 100 times larger than that of the core-cladding. All cracks appear visually identical and propagated successfully along the length of each waveguide as shown in Fig. 4c, while end facet cross sections verify the cleave covers the entire edge of the waveguide. It also partially wraps above and below the core, as might be expected intuitively from the non-zero but weaker stress field in those regions, giving an approximate total height of 15$\pm 1$ μm.

To confirm that more complex geometries can be produced using this process, Fig. 4d, e show cracks following sinusoidal and stepped 45$^\circ$ angle paths. These are intended to demonstrate the localised cleaving as a more general micromachining technique, rather than only for enhancing waveguides. Note in Fig. 4e the crack is seen to cross over from one side of the multiscan to the other; this occurs if the induced stress is too high on both sides of the waveguide, although here the higher stress at the corners due to segmented multiscan is also a contributing factor. For waveguides, such a crossing across the core would be lossy, but can be inhibited by adjusting the writing beam power according to the geometry or optimising the multiscan path to minimise high stress points.

Analysis of writing power $P_w$ influence on microcrack formation for a straight waveguide configuration in Fig. 4f indicates an optimum range 9.5 – 10.0 mW (pulse energy $E_p=47.5$ – 50 nJ) leads to consistent formation of microcracks on the desired side of the waveguide. Reducing $P_w$ to 9.0 mW, cracks start to form less predictably with a reduced height or not all, while at $P_w=8.5$ mW microcrack formation is completely suppressed. On the other hand, for higher powers of $P_w=10.5$ mW, the microcrack always forms but sometimes on the wrong side, and near the end facets the crack tends to widen which reduces the waveguide’s modal width. While obviously undesirable in our situation, this effect could however be exploited to form modal tapers.

For some applications it may be advantageous to form cracks on both sides of the waveguide, such as to further enhance modal confinement. However, once a crack begins to propagate on one edge, the local stress is reduced and insufficient to form a crack on the opposite waveguide edge. To create the second crack, another multiscan can be written to raise stress on the opposing side, followed by polishing. It is worth noting that in our tests, the waveguides are typically written with 80–100 μm spacing, so the formation of each waveguide crack does not interfere with the stress profile of its neighbours.

Multiscan line order was also investigated with straight waveguides in Fig. 4g. Inverting the order forces the crack to form on the other edge of the waveguide, as expected. However, if the multiscan begins from the center and scanlines alternate consecutively left and right, the symmetric stress forms the crack with equal chance on either side. Correct scanning order is thus straightforward but crucial for accurate placement of the crack.

### Experimental waveguide bends

**Figure 5:**
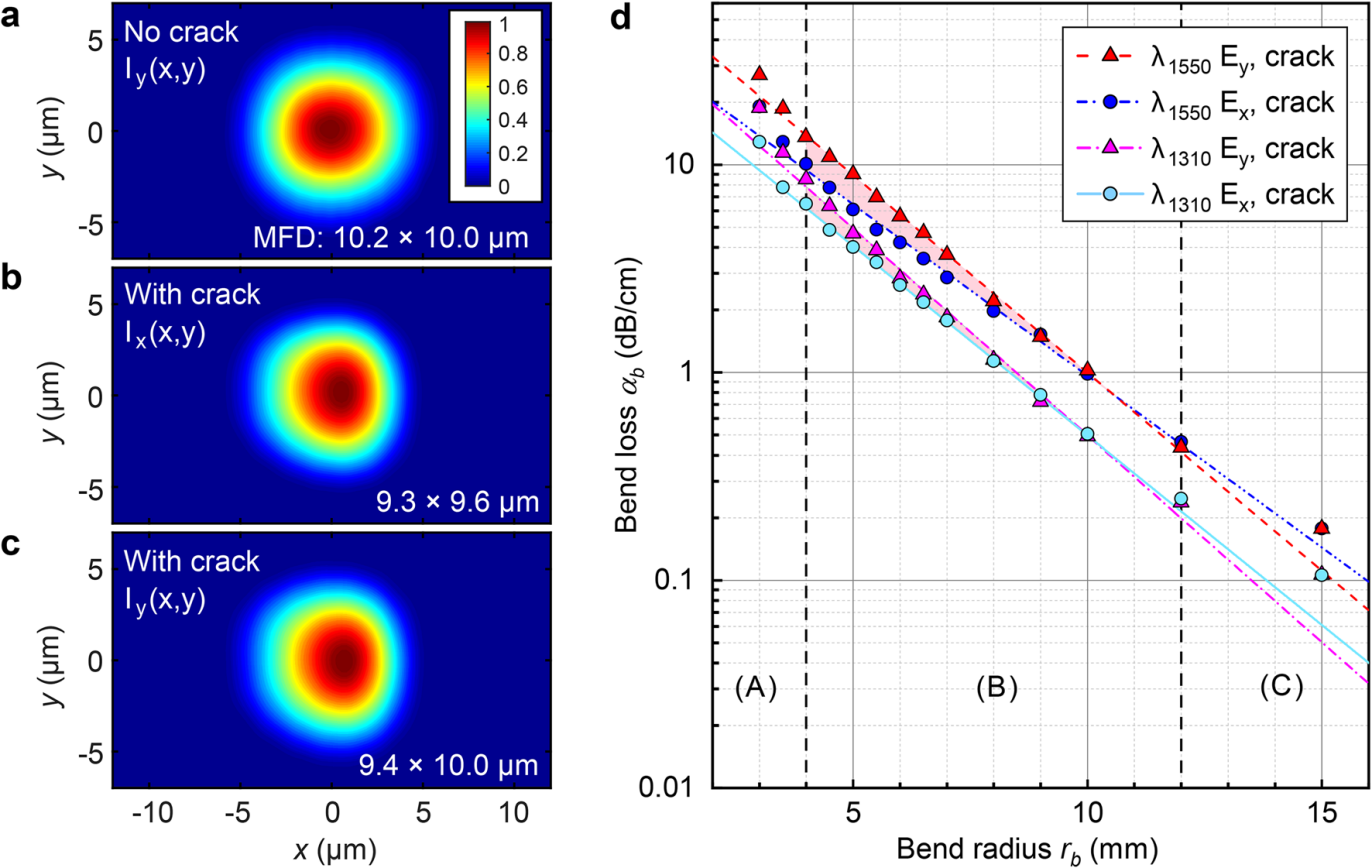
Experimentally measured waveguide mode profiles and bend loss. Mode intensity profiles for straight waveguides (**a**) without microcrack for *y* polarization (**b**) with microcrack for *x* polarization and (**c**) *y* polarization. Crack is on right side of waveguide and peak intensity normalised to unity. The 1/$e^2$ mode field diameters in the *x* and *y* dimensions are labelled. (**d**) Measured bend loss $\alpha _b$ against bend radius $r_b$, for both polarizations at $\lambda =1550$ nm and 1310 nm. Shaded region denotes polarization-dependent loss. Vertical lines divide the bend radius into 3 ranges A, B and C, where dominant losses are excessive confinement loss, bend loss, and scattering losses, respectively.

With all microcracks verified to have formed correctly, the waveguide mode profiles and bend losses were then characterised (Fig. 5). For comparison, Fig. 5a shows the mode for an uncracked straight waveguide, i.e. written with equivalent parameters but with the end faces polished before inscription. Without the microcrack, the $1/e^2$ mode field diameter is $10.2\times 10.0 \pm 0.1$ μm in both dimensions, but with the microcrack, it narrows by 6 – 7% in the *x* direction for both polarizations, with visible asymmetry due to increased modal confinement from the large $\Delta n$ of the crack interface on the right side.

The measured bend losses for the microcrack waveguides at $\lambda = 1.55$ and 1.31 μm in Fig. 5d show that the crack indeed successfully reduces loss. For $\lambda =1.55$ μm, 1 dB/cm is measured at a very tight radius of $r_b=10$ mm for both polarizations (for comparison, this surpasses the lowest loss reported until now of 2.7 dB/cm for this wavelength and bend radius^16^). For $\lambda =1.31$ μm, the stronger modal confinement provides 1 dB/cm at $r_b=8.5$ mm for both polarizations.

As expected from simulations, some polarization dependent loss is present (the *y* polarization exhibiting higher losses) and increases with decreasing bend radius as predicted. For example, at 1.55 μm wavelength, 3 dB/cm is measured at $r_b=6$ mm for the *x* polarization, but at $r_b=8.3$ mm for the *y* polarization.

For further analysis, we divide the bend radius range into 3 regimes, labelled A, B and C in Fig. 5d and demarcated by the vertical dashed lines. In region B for $r_b$ between 4 and 12 mm, the logarithmically plotted dB/cm loss follows a linear trend against $r_b$, which is typical of waveguide bend loss in general^32,33^. The gradient evaluates to $\alpha _b$ falling a decade with every 6 mm and 5.5 mm increase of $r_b$, for the *x* and *y* polarizations, respectively.

In region A for tight bends $r_b<4$ mm, the loss exceeds this trend as modal confinement is far too weak. Meanwhile for large bend radii in region C, the trend is invalid since the loss approaches a lower limit dictated by the propagation loss of a straight waveguide (0.10 and 0.15 dB/cm for $\lambda =1.31$ and 1.55 μm, respectively). This is substantiated by tests comparing straight cracked and uncracked waveguides showing no measurable difference in their propagation losses per unit length, suggesting negligible scattering arises from the crack. The propagation loss is primarily attributed to scattering losses from small variations in the induced index change.

Compared with SMF-28 fiber, the *x* polarization bend loss here is in fact slightly lower than that of the fiber for curves tighter than 9 mm radius, e.g. at $r_b = 7$ mm, $\alpha _b=3.2$ dB/cm for SMF-28^34^ versus 2 dB/cm for the waveguide. For looser bends $r_b>9$ mm however, the fiber loss is lower due to its far smaller propagation loss in the straight guidance limit of 0.02 dB/km.

To investigate long term stability, several samples were re-characterised after one year in storage at room temperature in a sealed membrane box, which confirmed no observable differences in bend loss or crack dimensions occurred. Stability against thermal change was also confirmed by heating one sample to 400 $^\circ$C for 4 h on a hot plate and allowing it to cool down at room temperature, without any change in transmission properties before and after heating. These tests imply the embedded cracks possess both long-term stability and resistance against thermal cycling up to at least 400 $^\circ$C.

## Discussion

These precision microcracks and their repeatable fabrication process have not been previously reported, and the closest structures offering the same large $\Delta n$ are microvoids^18^ and microchannels^35^. Voids are essentially 1D structures, while microchannel fabrication is time consuming and requires etching steps which cannot be controlled to the same precision. As the microchannel width is much larger than the microcrack, it is unsuitable for compact integration. Aside from the application demonstrated here, the ability to locally cleave glass therefore introduces many possibilities not previously possible, such as improving light confinement for guided wave optics, e.g. tapers to reduce mode size for integrating different components, as well as partial reflector surfaces embedded in glass such as a beamsplitter or resonant cavity. Longitudinally periodic cracks could be utilised for strong long period gratings, or efficient diffraction optics. More generally, the cracks could also be adopted for glass microstructuring, for example, by pre-cleaving glass to define and thus more selectively etch microchannels used in optofluidic chips.

To summarise, we introduced a technique to fabricate microcracks embedded in glass with excellent control and reliability. Femtosecond laser pulses were used to induce an asymmetric stress pattern to guide microcrack propagation, allowing compact and precise photonic device integration. As an example application, we inscribed 90$^\circ$ curved waveguides with microcracks on their outer bend edge to enhance modal confinement, which suppressed radiative bend loss to as low as 1 dB/cm at a bend radius of 10 mm for $\lambda =1.55$ μm, without causing additional scattering. This is the lowest reported loss for femtosecond written bends in silica, to the best of our knowledge. The crack and waveguides are mechanically stable over a year after fabrication, as well as being thermally stable up to at least 400 $^\circ$C. The ability to controllably cleave microcracks adds to the ever-growing arsenal of ultrafast laser material processing techniques, towards new potential optical device designs and functionality.

## Methods

### Fabrication of curved waveguides

Arrays of curved waveguides following a 90$^\circ$ arc with bend radii $r_b$ from 3 to 15 mm were inscribed on a $2\times 2\times 0.2$ cm fused silica substrate sample (UVFS C7980 0F, Altechna Ltd.), 0.5 mm below the surface. The waveguide at each bend radius was repeated 4 times, separated by 100 μm. Each waveguide was inscribed with a $10\times 10$ μm cross section to roughly mode-match with commercial standard SMF-28 fiber at 1550 nm wavelength.

The femtosecond laser writing system is shown in Fig. 1a. To inscribe the waveguides, 200 fs pulses at a wavelength of $\lambda =515$ nm, generated as a second harmonic from a 200 kHz 1030 nm Yb:KGW laser pump (PHAROS, Light Conversion Ltd.), were focused by a 0.4 NA objective lens ($\times$25 0.4NA PLAN, Leitz Wetzlar) onto the sample. The average power was 9.5$\pm 0.1$ mW (pulse energy 47.5 nJ). The sample was translated by a computer controlled two-axis transverse stage (ANT130-160-XY, Aerotech Ltd.), while the objective height was positioned by a vertical stage (ANT130LZS, Aerotech Ltd.). A circularly polarized writing beam was chosen to induce a higher index change^14^ and inhibit formation of Type II modification nanograting damage^36^, which would be scattering and introduce additional PDL.

All waveguides were inscribed using the multiscan method^37^ with a scanline separation of $s=200$ nm. Each scanline was written at a high pulse density of $D=3\times 10^5$ pulses/mm and pulse energy $E_p\approx 50$ nJ. Most importantly, the first scanline must begin on the inner bend edge, so that as subsequent scanlines are written adjacently, the accumulation of stress leads to higher stress on the outer bend, where the crack will form.

After inscription, the end facets were exposed by lapping approximately 200 μm with calcined aluminium oxide suspension (successively, from 3 to 1 to 0.3 μm particle size) in order to release and propagate the crack. Finally, polishing using SF1 polishing fluid (Logitech Ltd.) was undertaken to achieve an optical finish suitable for direct fibre coupling during characterisation.

In addition, straight waveguides of length 1 cm were fabricated using the same procedure to check the nominal propagation loss ($r_b=\infty$). To produce the uncracked equivalent waveguides (Fig. 5a), the sample end faces were pre-polished before waveguide inscription, so the crack is not formed.

### Retardance measurements

For the retardance and stress measurements, the test waveguide-like structures in Fig. 2 were fabricated using a Gauss-Bessel beam, by inserting an axicon with a 179$^\circ$ apex angle approximately 1 meter before the 0.4 NA objective lens; the set up is otherwise the same as Fig. 1a. Waveguides were written by multiscan at pulse energies of 0.45, 0.55 and 0.65 mJ, corresponding to powers of 90, 110 and 130 mW ($\pm 0.05$ mW), respectively. The modified region cross-section width and height are 20 μm and 100 μm, respectively.

After fabrication, the retardance profiles were recorded using an optical microscope system with a $\times 100$ objective lens in conjunction with a VariLC liquid crystal device controlled by OpenPolScope software. The retardance was measured in the same direction as that of the laser writing beam during fabrication, i.e. perpendicular to the plane of the waveguide array. The principal stress difference $\Delta \sigma$ was then calculated by the relation ${\Delta \sigma = R/(C T)}$^38^, where $C=3.55\times 10^{-12}$ Pa$^{-1}$ is the photoelastic coefficient between the two principal stresses in silica, and $T\approx 100$μm the waveguide height.

### Waveguide characterisation

To characterise waveguide loss and mode field profiles, continuous-wave light from a 1.55 μm or 1.31 μm single-mode laser diode source was passed through a polarizer and half-waveplate to control polarization angle, then directly butt-coupled to the waveguide input facet by polarization-maintaining fiber. Light from the waveguide output was then collected and focused by an objective onto either a power meter or profiling camera (MicronViewer 7290A, Electrophysics), to obtain loss or near-field mode profile measurements, respectively. In both cases, an iris was inserted before the detector to block scattered light and minimise ambient illumination noise. All the fibers, and the waveguides themselves, are single-moded at both the interrogation wavelengths used.

The method described above will measure the total insertion loss $\alpha _i$, from which the bend loss $\alpha _b$ can be extracted:

1 $$\begin{aligned} \alpha _i = (\alpha _b + \alpha _m)L + \alpha _{f1} + \alpha _{f2} + \alpha _c \end{aligned}$$

where $L=\pi r_b/2$ is the waveguide length, $\alpha _m$ the material loss coefficient, $\alpha _{f1,f2}$ the Fresnel reflection losses at the input and output facets (totalling 0.26 dB), and $\alpha _c$ the input coupling loss due to fiber-waveguide mode mismatch. Based on overlap integrals, $\alpha _c\approx 0.05$ dB, and $\alpha _m$ is assumed negligible. Note that $\alpha _b$ represents the loss per unit length around the bend, i.e. includes radiative losses as well as any scattering propagation losses (for a straight waveguide, $\alpha _b$ would simply be the propagation loss per unit length).

### Modelling

To simulate the bend loss and mode profiles for the curved waveguides, an axisymmetric 2D mode analysis was performed in a commercial finite element method solver software (COMSOL 5.3). The waveguide core was modelled as a $10 \times 10$ μm square cross section with a uniform step-index profile and core-cladding index difference $\Delta n = n_1 - n_2$ of $4.1\times 10^{-3}$, based on previous work comparing the experimental and simulated mode field diameter of waveguides. The fused silica substrate (cladding) index $n_2$ was calculated according to the Sellmeier equation^39^. The crack profile was modelled as a 30 nm wide, 15 μm high rectangle with an index of $n_c=1$, positioned against the outer bend edge of the core. The width of the crack $w_c$ also affects the bend loss to some extent. From microscopy images, it is not possible to resolve the crack width beyond an upper limit of $w_c<200$ nm, and unfortunately SEM imaging does not provide a suitable image since the crack at the facet is partly filled during polishing, and in any case is not subject to the same stresses as the part of the crack within the sample. For this reason, in these simulations the crack width was estimated to be 30 nm, based on comparing the simulated and observed experimental trends. To absorb the field radiated from the bend, a perfectly-matched layer was added to the outer bend region of the simulation space, and solution of the modal eigenvalue equations yields complex effective indices from which the bend loss coefficients were extracted.

## Data Availability

The data in this work is available upon request.
